# Efficacy and Mechanisms of Butyric Acid Derivatives as Feed Additives in Weaned Piglet Nutrition: A Review

**DOI:** 10.3390/antiox15070805

**Published:** 2026-06-27

**Authors:** Weican Zhang, An Tao, Xingping Chen, Xin Li, Tiande Zou, Jun Chen, Jinming You

**Affiliations:** Jiangxi Province Key Laboratory of Animal Nutrition and Feed, College of Animal Science and Technology, Jiangxi Agricultural University, Nanchang 330045, China; weicanzhang@stu.jxau.edu.cn (W.Z.); taoan2023@163.com (A.T.); cxp0315@jxau.edu.cn (X.C.); lixin@jxau.edu.cn (X.L.); tiandezou@jxau.edu.cn (T.Z.)

**Keywords:** anti-inflammatory effects, antioxidant, butyric acid, functional feed additive, gut microbiota, intestinal health, weaned pigs

## Abstract

Early weaning can disrupt the intestinal function and microbial community balance of piglets, and trigger inflammation and oxidative stress, thereby affecting their production performance. In recent years, butyric acid has gained considerable interest as a functional feed additive. However, practical limitations such as its pungent odor and low absorption efficiency in the digestive tract have led to the development of more stable forms, including sodium butyrate, coated butyrate, and butyrate glycerides, etc. Research has shown that butyric acid and its derivatives can serve as effective feed additives by enhancing pigs’ resistance to pathogenic colonization, stabilizing the intestinal microbiota, and alleviating oxidative stress to mitigate challenges such as weaning stress and pathogenic infections. This review systematically highlights the role of butyric acid and its derivatives as dietary supplements for weaned piglets. Importantly, it underscores the potential of butyric acid and its derivatives may contribute to antibiotic-reduction strategies in weaned piglet nutrition, while also highlighting the need for optimized supplementation strategies and further investigation into synergistic effects with other feed additives. This review aims to offer both theoretical and practical insights for the application of butyric acid in weaned piglet nutrition.

## 1. Introduction

In-feed antibiotics have long been used to enhance growth performance and prevent diseases of weaned piglets. However, excessive antibiotic use exacerbates bacterial resistance, posing a huge threat to both animal and human health [1,2]. In response, the European Union banned the use of antibiotics as growth promoters in animal feed in 2006. Since 2020, China has also prohibited the use of antibiotics for growth promotion in animal feed. Consequently, there is an urgent need for safe and effective alternatives for weaned piglets.

Butyric acid exhibits multiple biological functions, including antibacterial, antioxidant, and anti-inflammatory properties, as well as maintenance of intestinal function [3,4,5]. Generally, the administration of butyric acid can reduce the pH of gastrointestinal contents, and the magnitude of this effect also depends on several factors, including its form, supplemental dose, site of release, and the buffering capacity of the diet. Butyric acid can effectively inhibit pathogenic bacterial colonization while enhancing immune function and growth performance. In this regard, it holds potential application prospects as an alternative to antibiotics in swine feed [6,7]. However, butyrate has limitations, including its unpleasant odor and absorption issues in the upper digestive tract, which hinder its ability to exert its full effects [8]. In weaned piglet nutrition, it is typically formulated into products with more stable chemical forms, such as sodium butyrate, coated butyrate, and butyrate glycerides. A comparison of main characteristics of different butyric acid forms is presented in Table 1. Although the role of butyric acid as a feed additive has been documented in existing literature, systematic reviews on its effects on piglets during the weaning stage remain limited.

As such, this review updated current knowledge on the use of butyric acid and its derivatives in weaned piglets. For the section on butyric acid application in weaned piglets, a systematic search was conducted to identify relevant articles published before May 2026 in the Web of Science, PubMed, Scopus and Google Scholar databases. The search terms included “butyric acid + weaned piglets”, “tributyrin + weaned piglets”, “butyrate glycerides + weaned piglets”, and “sodium butyrate + weaned piglets”. Studies focusing on other swine phases were excluded, with only research involving weaned piglets included to ensure relevance to butyric acid application in this specific group. This review combines in vivo and in vitro experiments to analyze and improve the possibility of using butyric acid and its derivatives in weaned piglets.

## 2. The Sources and Main Biological Functions of Butyric Acid

Butyric acid, a short-chain fatty acid (SCFA), is primarily generated via the fermentation of indigestible carbohydrates by intestinal microorganisms. As a key metabolite produced by the gut microbiota, it has emerged as a potential biomarker for evaluating overall physiological status [18,19]. Butyric acid serves as the principal metabolic substrate for colonic epithelial cells, with the majority of butyric acid being metabolized by colonocytes [20]. *Lactiplantibacillus plantarum* has been demonstrated to enhance butyric acid production by upregulating genes involved in lactate utilization and butyrate synthesis [18], while its combination with *Clostridium tyrobutyricum* can elevate intestinal butyric acid levels [21]. Additionally, the microbial community exhibits a close association with the composition of short-chain fatty acids in rectal contents: studies have confirmed that *Prevotella* increases the abundance of butyric acid in rectal contents, whereas *Akkermansia* decreases it [22]. *Anaerostipes hadrus*, a Gram-positive anaerobic bacterium, is distinctly characterized by its capacity for butyric acid production [23], can utilize lactate and acetate generated by *Bifidobacterium* and *Lactobacillus* through co-metabolism to convert them into butyric acid [24].

**Figure 1:**
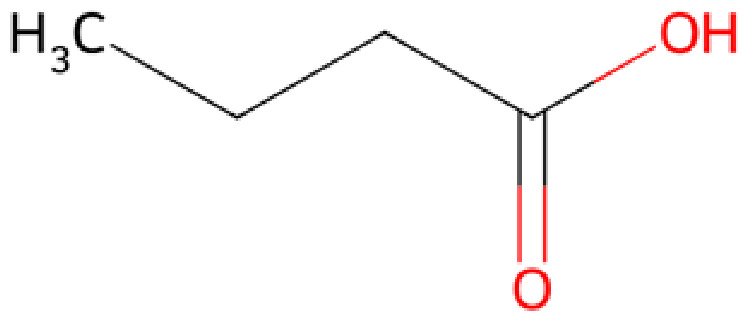
Chemical structure of butyric acid.

Butyric acid is a monocarboxylic acid with the molecular formula CH_3_(CH_2_)_2_COOH (Figure 1). At room temperature, it is a colorless liquid with a pungent odor reminiscent of rancid oil. This acid is volatile, unstable in aqueous solutions, and undergoes rapid decomposition [25,26]. Naturally occurring in foods such as butter and milk, the predominant source of butyric acid in the human body is microbial fermentation of carbohydrates and proteins in the large intestine, yielding a C4 fatty acid [27]. Butyric acid exhibits potent antioxidant properties, contributing to its health benefits [28]. Additionally, as the primary energy source for colonocytes, butyric acid supports intestinal homeostasis. By providing energy to intestinal epithelial cells, it enhances metabolic activity, strengthens protective functions, and mitigates intestinal disorders [29,30]. Emerging evidence further suggests its anti-inflammatory, antibacterial, and antiviral potential [31]. The following sections will systematically summarize the research progress on butyric acid and its derivatives in swine nutrition, with a primary focus on weaned piglets.

## 3. Research Progress on Butyric Acid and Its Derivatives as Functional Feed Additives in Weaned Piglet Nutrition

Early weaning of piglets is a practice in intensive pig production, yet it is associated with multiple stress-inducing factors. These include weaning stress, nutritional and environmental stress, high susceptibility to diseases, as well as underdeveloped digestive and immune systems [32]. Such stressors lead to compromised growth performance and overall health in piglets [33,34]. The use of butyric acid in weaned piglet nutrition represents a promising nutritional strategy to address the critical challenges encountered during early development.

### 3.1. Effects of Butyric Acid and Its Derivatives on Growth Performance of Weaned Piglets

Weaning is a crucial period in the life cycle of pigs, often accompanied by significant physiological stress. Butyric acid plays a crucial role in promoting piglet growth, particularly after weaning, as documented in numerous studies. Wan et al. [12] investigated the impact of sodium butyrate at different doses of 1000 and 2000 mg/kg for 28 days on weaned piglets, and found that adding two different doses of sodium butyrate led to an increase in average daily feed intake (ADFI) by approximately 150 g on average compared with control. In addition, it was observed that when the dosage reached 2000 mg/kg, average daily gain (ADG) was elevated, while feed conversion ratio (F/G) significantly decreased [12]. Similarly, Long et al. [35] indicated that supplementing weaned piglet diets with 2000 mg/kg organic acid containing butyric acid remarkably increased ADG, while significantly decreased the diarrhea rate and F/G. Another study discovered that the addition of 1000, 1500, or 2000 mg/kg encapsulated sodium butyrate to piglets at 20–69 days of age exhibited positive effects on the growth performance as manifested by a decrease in F/G [14]. Analogously, Correia et al. [10] reported that adding 1000 or 1500 mg/kg sodium butyrate to piglets at 21–45 days of ages significantly increased the ADG. In addition, it can be observed that the final body weight significantly increased when the piglets were fed with 1000 mg/kg sodium butyrate. Furthermore, Dong et al. [17] explored the effects of tributyrin on weaned pigs. The findings demonstrated that the inclusion of 1000 mg/kg tributyrin in diet could improve growth performance. Additionally, the authors observed body weight significantly increased at 17 and 20 days and increased spleen weight [17]. However, a study has shown that when exposed to a 2 mL dose (5 × 10^8^ CFU) of *Salmonella* Typhimurium, adding the inclusion of 2100 mg/kg sodium butyrate in diet does not affect growth performance in piglets [13]. These discrepancies may be explained by differences in rearing conditions: the piglets in this study were challenged with *Salmonella* Typhimurium, whereas the improved growth performance reported in the other study was observed in piglets not subjected to a stress model. These results further suggest that sodium butyrate supplementation may need to be increased when weaned piglets are exposed to pathogenic infections. Yet, microbial-derived butyrate plays a crucial role in the health of weaned piglets. This study found that ADG, ADFI, and final body weight increased as the growth stage extended [36].

More importantly, butyric acid has been shown to promote growth in weaned piglets under special physiological conditions, stress, and infection status. Li et al. [37] investigated the effects of low protein diets supplemented with 2000 mg/kg sodium butyrate. The authors found that the ADFI of weaned piglets significantly increased [37]. In addition, Gu et al. [16] found that adding 2000 mg/kg tributyrin to piglet diets containing antibiotics significantly increased ADG, ADFI, and final body weight. In a 28-day trial conducted by Weber et al. [38], when challenged with LPS (25 μg/kg of BW, *E. coli* serotype O55:B5), the addition of with 2000 mg/kg sodium butyrate did not exhibit any impact on the growth performance in weaned piglets. Weaned piglets are highly susceptible to enterotoxigenic *Escherichia coli* (ETEC) infection, which leads to diarrhea and growth restriction [39,40]. Subsequently, Kovanda et al. [15] reported that adding 1000 mg/kg butyrate glycerides to the diets of piglets weaned at 21–24 days of age improved the health condition and reduced the diarrhea rate of ETEC K88-infected weaned piglets in a 14-day feeding trial. When subjected to ETEC challenge, piglets supplemented with butyric acid exhibited significantly greater growth performance and improved health status compared with the control group.

Additionally, recent studies have investigated the synergistic effects of sodium butyrate and other feed additives. According to the study conducted by Hanczakowska et al. [41], there were no observed effects on growth performance of weaned pigs when a dietary supplement of 3000 mg/kg sodium butyrate was combined with 10,000 mg/kg glutamine or 10,000 mg/kg glucose separately. However, Diether et al. [42] observed that the ADG of piglets significantly increased when they were fed with 2000 mg/kg MCOA (a blend of medium chain fatty acids, organic acids, slow release C12, target release butyrate and a phenolic compound). In another study, Zhen et al. [43] reported that supplementing weaned piglet diets with a mixture of 2000 mg/kg sodium butyrate and different proportions of nicotinamide improved the growth performance of weaned piglets. Significantly, the authors found that the growth performance of weaned piglets improved when 40 mg/kg nicotinamide and sodium butyrate were used in combination [43]. This effect was indicated by decreased F/G as well as increased ADG. More interestingly, the diarrhea condition of the piglets improved significantly when 2000 mg/kg sodium butyrate and 160 mg/kg niacin were added together [43]. Dietary supplementation with incremental levels of 350, 700, 1050 mg/kg of sodium butyrate in benzoic acid-containing diets induced distinct growth response patterns in weaned pigs: ADG and final body weight showed a quadratic increase under experimental conditions at the University of Illinois Urbana-Champaign. At the University of Arkansas, ADFI increased significantly when the piglets were supplemented with 350 mg/kg sodium butyrate [44]. The coated sodium butyrate and butyrate glyceride are more stable in structure, have almost no odor, and offer better palatability. After being added, it can improve the growth performance of weaned piglets. In summary, the combination of sodium butyrate and other substances may promote the growth performance of weaned piglets, but the specific mechanism of combined effects requires further research.

### 3.2. Effects of Butyric Acid and Its Derivatives on Antioxidant Status of Weaned Piglets

The antioxidant activity of butyric acid is one of its primary biological properties, which has been demonstrated in weaned piglets. Oxidative stress exerts a dual role during piglet infection: the free radicals it generates may cause tissue damage during inflammation, but they may also resist microbial invasion. Studies have indicated that nutritional intervention could be a potential approach to combat diseases [45].

In an in vitro study, it was revealed that IPEC-J2 cells with 440 mM of sodium butyrate for 2 days resulted in a significant reduction in oxidative damage [46]. More specifically, Ma et al. [46] observed that sodium butyrate treatment increased the activities of superoxide dismutase (SOD), glutathione peroxidase (GSH-Px), and glutathione (GSH) levels while decreasing the level of malondialdehyde (MDA). Similarly, a recent study revealed the potential of 2000 mg/kg chemically protected sodium butyrate treatment for 28 days in ameliorating oxidative damage in weaned piglets [12]. The treatment with sodium butyrate resulted in a decrease in MDA level while simultaneously enhancing total antioxidant capacity (T-AOC), SOD, GSH-Px, and catalase (CAT) activities in serum [12]. The Nrf-2-Keap1 system, as an antioxidant stress mechanism, is a defense system to maintain cellular homeostasis [47]. Nrf2 dissociates from Keap1 when cells are exposed to electrophilic and oxidative stress, allowing its translocation to the nucleus and subsequent upregulation of antioxidant genes through ARE binding [48]. Furthermore, the sodium butyrate treatment increased colonic *Keap1*, *Nrf-2*, *CAT* and *SOD1* mRNA levels, thereby activating the endogenous antioxidant defense system of weaned piglets [12]. Additionally, as reported by Li et al. [37], supplementing a low-protein diet with 2000 mg/kg sodium butyrate improved the antioxidant status of piglets, including increasing GSH-Px activity in plasma. Butyrate, an inhibitor of histone deacetylases (HDACs), can upregulate antioxidant genes by inhibiting the binding of NF-κB to HDAC [49]. In another study, supplementing weaned piglets with 5% dietary-derived butyrate alleviated weaning stress; as for the mechanism of action, the administration of butyrate activated the MyD88-NF-κB signaling pathway [50]. Notably, this alleviation was evident from a decrease in the levels of MDA and reactive oxygen species (ROS) in serum as well as increased activities of SOD and T-AOC in serum [50]. More interestingly, another study found that administering a mixture of organic acids containing butyric acid at a dose of 2000 mg/kg led to a reduction in oxidative damage. This reduction was observed in the level of hydroxyl radicals in serum [35].

DON can reduce T-AOC in weaned piglets, thereby inducing oxidative stress. Evidence regarding the ability of sodium butyrate to alleviate the oxidative damage caused by DON can be obtained from the work of Zong et al. [28], who demonstrated that the beneficial effects of 2000 mg/kg sodium butyrate on piglets under DON challenge. This effect can be observed from a decrease in MDA level, while an increase in T-AOC, SOD, GSH-Px activities through histone acetylation, thereby reducing oxidative stress [28]. Marchiori et al. [51] discovered that when weaned piglets consume dietary supplements containing sodium butyrate or butyrate glycerides, oxidative damage is reduced. This was indicated by reduced lipid peroxidation product, while increased serum activities of SOD, glutathione s-transferase (GST). The study also found that butyric acid glycerides significantly increase the serum GPx activity [51]. Moreover, the antioxidant status in the liver and intestines was also significantly enhanced [51]. Sodium butyrate and its derivatives can improve the antioxidant status of weaned piglets throughout the body, from serum to liver and intestines. Based on the existing evidence, it is believed that butyric acid and its derivatives have significant potential as antioxidants, which can enhance the antioxidant capacity of piglets and reduce oxidative damage. Nevertheless, further investigations are required in vitro and ex vivo to authenticate the specific mechanism of action of butyric acid, which is of great significance for promoting the research and application of butyric acid in piglet nutrition.

### 3.3. Effects of Butyric Acid and Its Derivatives on Inflammation Modulation of Weaned Piglets

Inflammation has been reported as a cost to productivity in the swine industry and serves as the basis for allocating nutritional resources between growth and survival in pig production [52]. Inflammatory responses are closely associated with animal diseases and can lead to reduced productivity or survival rates in pigs [53]. Therefore, it is imperative to explore strategies to enhance the immunity of weaned piglets and control inflammatory responses. Sodium butyrate possesses the function of regulating inflammatory responses and has been proven to exert health promoting effects on weaned piglets [54]. A dose of 2000 mg/kg of chemically protected sodium butyrate supplementation markedly inhibited the inflammatory response [12]. This reduction was evident from a decrease in the levels of tumor necrosis factor-α (TNF-α) in the serum, whereas an increase in the levels of interleukin 10 (IL-10) in the serum [12]. Notably, Wan et al. observed that sodium butyrate treatment elevated colonic mRNA levels of *IL-10*, while reduced colonic mRNA levels of *IL-1β* [12]. This indicates that the anti-inflammatory effect of sodium butyrate is not localized but systemic, which guides subsequent research on combating systemic infections. Additionally, microbially derived butyrate can maintain the balance between cell apoptosis and proliferation by reducing the mRNA and protein expression of TNF-α and Interferon-γ to alleviate intestinal inflammatory responses [36].

The role of butyrate is also crucial under stress and infection conditions. The supplementation of sodium butyrate has also been found to ameliorate inflammation induced by a 10 μg/mL LPS challenge in the IPEC-J2 cells [55]. Farkas et al. [55] reported that treating IPEC-J2 cells with 220 mM sodium butyrate for one day resulted in an alleviation of the inflammation response when challenged with 1 μg/mL LPS from *Salmonella enterica* serovar Typhimurium. Additionally, this was indicated by decreasing the levels of interleukin 8 (IL-8) in IPEC-J2 cells [55]. This reflects that butyrate exhibits comprehensive anti-inflammatory performance in the most realistic scenarios. Moreover, Han et al. [56] assessed the beneficial effects of butyrate on weaned piglets under LPS challenge. It was found that the administration of 3000 mg/kg coated butyrate, significantly reduced the levels of interleukin 1β (IL-1β) and interleukin 6 (IL-6) in the jejunum after a 100 μg/kg LPS (from *E. coli* serotype 055:B5) challenge [56]. Furthermore, it was observed that the levels of IL-10, IL-13, and transforming growth factor-β (TGF-β) in the jejunum were markedly increased. In addition, as for the mechanism of action, research has shown that the addition of 3000 mg/kg coated butyrate inhibits the NF-κB/HIF-1α signaling pathway in weaned piglets, thereby improving the jejunal inflammatory state [56]. Under 25 μg/kg LPS (from *E. coli* serotype 055:B5) challenge, sodium butyrate can regulate the inflammatory response of piglets by reducing the mRNA levels of *IL-6* in Longissimus muscle [38]. As reported by Gu et al. [16], adding 2000 mg/kg tributyrin enhanced the anti-infection ability of piglets by reducing the levels of IL-6 in the plasma after LPS infection. In addition, Tian et al. [57] reported that the dietary addition of 5000 mg/kg glyceryl butyrate alleviated the inflammatory response of weaned piglets induced by the ETEC (serotype O149: K91: K88ac) challenge at 5 × 10^10^ CFU. This was indicated by decreased jejunal levels of IL-6, IL-1β and TNF-α, as well as a decrease in levels of IL-6 in the ileum. In summary, these studies demonstrate that butyrate modulates the inflammatory responses of weaned piglets under different scenarios. However, further research is required to support the implementation of sodium butyrate as a treatment for inflammation associated with weaned piglets.

### 3.4. Intestinal Morphology

Impairment of intestinal morphology in piglets is a challenge during the post-weaning period, which affects their normal digestion and nutrient absorption [29]. It has been reported that butyric acid can improve intestinal morphology and promote digestive absorption in piglets [58,59].

According to Long et al., 2000 mg/kg of mixed organic acid containing butyric acid significantly reduced the jejunal crypt depth (CD), while increased the jejunal and ileal the villus height/crypt depth (VH/CD) [35]. Similarly, Zeng et al. [60] demonstrated that adding 3000 mg/kg sodium butyrate to the diet of weaned piglets increased the ileal villus height (VH) and VH/CD, thereby improving ileal morphology. In addition, butyric acid glycerides have been proven to improve the intestinal morphology function of weaned piglets [51]. In an experiment conducted by Marchiori et al. [51], piglets that were supplemented with butyric acid glycerides had significantly improved intestinal morphology, as indicated by the decreased the CD and increased VH/CD [51]. Compared with free butyric acid, butyrate glyceride can specifically target intestinal segments, thereby improving the morphology of the intestine. In addition, Manzanilla et al. [61] clarified that dietary addition of 3000 mg/kg sodium butyrate during weaning notably increased the number of goblet cells in the colon. This ensures the integrity and proper arrangement of the crypt structures in the colon, as well as maintaining an appropriate thickness of the mucosal layer [61]. This is consistent with a study by Liang et al. [62], revealing that sodium butyrate promotes the polarization of macrophages to the M2 phenotype. M2 macrophages then secrete wingless/Int-1, which binds to the receptors on the precursor cells of goblet cells, activating the intracellular extracellular signal-regulated kinase signaling pathway, thereby promoting the differentiation of these cells into goblet cells. Furthermore, the butyrate derived from microorganisms was able to improve the morphology of the jejunum by significantly increasing the VH/CD in the jejunum and reducing the CD level in the second week of the experiment [36]. In addition, adding 3000 mg/kg sodium butyrate to the diet of weaned piglets improved the intestinal environment of piglets by promoting mucosal growth in the ileum and proximal and mid jejunum [63]. Similarly, in an experiment performed by Han et al. [56], the dietary inclusion of 3000 mg/kg coated butyrate for 21 days alleviated LPS-induced intestinal damage. This was indicated by increased the VH and VH/CD in the jejunum and ileum [56]. Liu et al. [64] exemplified that dietary addition of 2000 mg/kg of sodium butyrate increased the jejunal and ileal VH/CD, duodenal and ileal VH, and decreased the jejunal and colonic CD. Dong et al. [17] further found that adding 1000 mg/kg tributyrin to piglet diets significantly increased the duodenal and jejunal VH/CD, reduced the duodenal CD. Furthermore, the utilization of tributyrin resulted in an increase in duodenal mucosa thickness, muscle thickness, and villus surface [17]. It indicates that the maturation and integrity of the mucosal structure have been improved, thereby improving intestinal morphology [17]. The supplement of low-dose sodium butyrate in piglet diets has also been shown to improve intestinal morphology. As reported by Wang et al. [65], supplementing the diet of weaned piglets with 450 mg/kg sodium butyrate significantly increased the jejunal VH and VH/CD.

These findings suggest that sodium butyrate or its derived products have the potential to improve intestinal morphology in weaned piglets. However, the dosage should also be taken into consideration when utilizing sodium butyrate and its derivatives.

### 3.5. Intestinal Barrier Function

Maintaining normal intestinal physiological functions and homeostasis is crucial for the production efficiency and welfare of the swine industry [66]. Early weaning stress may lead to impaired physical barrier function, and increased intestinal permeability in piglets [67]. In an in vitro experiment, Ma et al. [46] found that the dietary supplementation of sodium butyrate could ameliorate intestinal barrier function induced by early weaning in IPEC-J2 cells. It was reported that the treatment of IPEC-J2 cells with 440 mg/kg sodium butyrate for 2 days improved intestinal barrier function [46]. This is manifested by the upregulation of the mRNA levels of *occludin* and *ZO-1*. The author holds that the intestinal barrier function has been enhanced and improved intestinal leakage [46]. A study by Feng et al. [63] found that supplementing with 2000 mg/kg sodium butyrate effectively upregulated the colonic and ileal mRNA levels of *claudin-3* and *occludin*, and the colonic mRNA levels of *ZO-1* was also upregulated. This increase indicates an enhancement in the barrier function [68]. Consistently, the administration of 1000 mg/kg sodium butyrate led to improvements in the intestinal barrier. These enhancements were indicated by reduced the levels of diamine oxidase and D-lactic acid in serum [69]. This manifests that the addition of sodium butyrate achieves this by reducing intestinal permeability, protecting the intestinal barrier function, and maintaining the integrity of intestinal epithelial cells [69]. Subsequent studies have also shown that sodium butyrate can improve intestinal barrier function. Liu et al. [64] observed that supplementing with 2000 mg/kg sodium butyrate significantly upregulated the ileal mRNA levels of *MUC1*, *PKC*, *ITGB* and *claudin-1*. As observed by Correia et al. [10], dietary supplementation with 1500 mg/kg sodium butyrate markedly upregulated the jejunal mRNA levels of *MCT1*, *SGLT1*, *occludin* and *ZO-1*, improving the intestinal barrier function of piglets.

Deoxynivalenol (DON) is a trichothecene mycotoxin produced by fungi of the genus *Fusarium*, which can cause impairment of intestinal barrier function in piglets [70]. Wang et al. [71] demonstrated that adding 2000 mg/kg sodium butyrate to weaned piglets could alleviate the intestinal barrier function damage caused by DON. This mechanism achieves this alleviation by modulating the NOD2/caspase-12 pathway to promote the assembly of intestinal host defense peptides [71]. The specific manifestation is an increase in the jejunal mRNA levels of *PMAP23* and *claudin-4* [71]. Additionally, Xue et al. [72] further found that supplementing piglet diets with 2000 mg/kg sodium butyrate could repair the intestinal barrier damage caused by DON by improving phosphoenolpyruvate carboxykinase 2-mediated mitochondrial function. Notably, the jejunal expression levels of *claudin-4*, *ZO-1* and *occludin* significantly increased. This indicates that sodium butyrate has great application potential in alleviating DON-induced intestinal barrier damage in piglets [72]. Rotavirus (RV) is one of the common viruses in large-scale pig production; it infects intestinal epithelial cells and causes dysfunction of the piglet mucosal mechanical barrier [73]. In the IPEC-J2 cell model, the treatment of 220 or 440 mM sodium butyrate led to an improvement in the epithelial barrier function [74]. This improvement was observed from significantly upregulating the mRNA level of *occludin*, *claudin-1* and *ZO-1*. The mechanism is activated through the adenosine monophosphate-activated protein kinase-nuclear factor erythroid 2-related factor signaling pathway via the G protein-coupled receptor 109A receptor [74]. This alleviated the downregulation of the antioxidant system caused by RV infection, thereby helping to restore the intestinal mucosal barrier function [74]. Compared with free butyric acid, sodium butyrate has a more stable structure and can avoid being released in the stomach and acting on the intestine, thereby improving the intestinal barrier function of weaned piglets. In summary, these findings indicate that sodium butyrate or its derivative products have the potential to promote intestinal barrier function by reducing intestinal permeability and regulating the expression of tight junction proteins.

### 3.6. Gut Microbiota

The intestinal microbiota is an indispensable component of piglet health, and organic acids, have been shown to exert a regulatory effect on this intricate ecosystem. The research reported by Long et al. corroborated this perspective [35], supplementing piglet diets with 2000 mg/kg mixed organic acids has been proven to maintain the stability of the intestinal microbial flora. Furthermore, supplementation with organic acids reduced the abundance of *Escherichia coli* and total anaerobic bacteria in the collected fecal samples [35]. Moreover, among organic acids, butyric acid has been demonstrated to regulate the stability of the intestinal microbiota in weaned piglets by promoting the proliferation of beneficial colonic bacteria and inhibiting the expansion of pathogenic bacteria [12]. As noted by Barba-Vidal et al. [13], the dietary addition of 2100 mg/kg protected sodium butyrate significantly decreased *Salmonella* shedders in feces and colonic digesta of weaned piglets. In another study, Xu et al. found [54] that oral administration of sodium butyrate at a dose of 150 mmol/L effectively increased the abundance and diversity of gastrointestinal microbiota. Specifically, it was observed that the abundance and diversity of *Acinetobacter*, *Actinobacillus*, *Facklamia*, *Globicatella*, *Kocuria*, and *Rothia* at the genus level increased [54]. Liu et al. [64] reported that supplementing piglet diets with 2000 mg/kg sodium butyrate resulted in an increase in the relative abundance of *Lactobacillus*, *Blautia*, *Eubacterium_rectale_group*, *Subdoligranulum*, *Coprococcus_3*, whereas a decrease in *Rikenellaceae_RC9_gut_group*, *Streptococcus*, *Prevotellaceae_NK3B31_group* at the genus level in the colon of weaned piglets. The authors concluded that this has facilitated amino acid metabolism and energy conversion processes in the colon [64]. Additional research has shown that adding 2000 mg/kg tributyrin to piglet diets resulted in a significant reduction in the colonic abundance of *Escherichia coli* after LPS infection [16].

**Figure 2:**
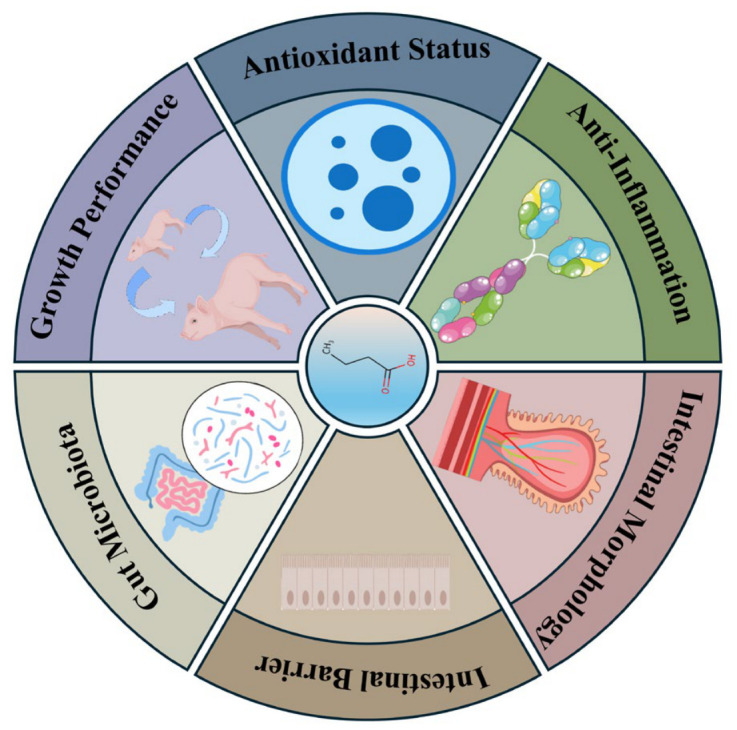
The graphic abstract of butyric acid and its derivatives applications in piglet nutrition. Due to its potential for anti-inflammatory, antibacterial and antiviral properties, adding butyric acid and its derivatives to the feed can effectively support growth performance and intestinal health in weaned piglets, and thus facilitate the application of butyric acid in piglet nutrition.

**Figure 3:**
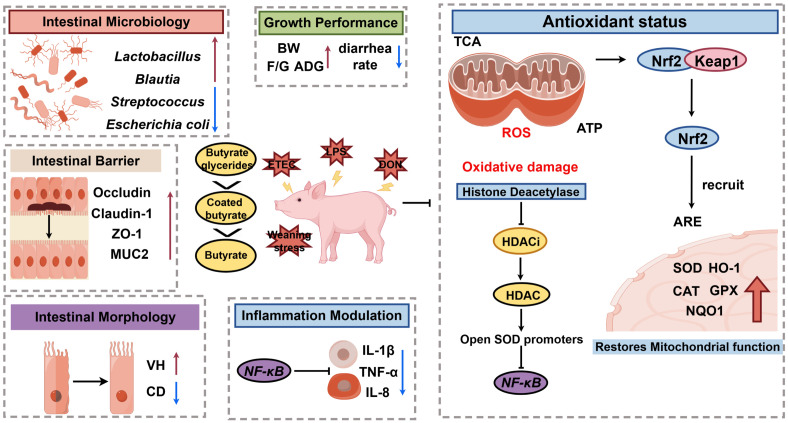
The detailed mechanism diagram of the effect of butyric acid and its derivatives on piglets under various stress conditions, including weaning stress, ETEC infection, LPS challenge, and deoxynivalenol (DON) challenge. The figure provides a detailed description of the mechanism by which butyric acid and its derivatives, as a feed additive, affect various indicators such as the growth performance, antioxidant capacity, and anti-inflammatory regulation of weaned piglets. Furthermore, the potential of butyric acid in terms of antioxidant activity is emphasized. This is achieved through pathways such as Nrf2-related signaling, histone deacetylase inhibition, improved mitochondrial function, reduced production of ROS driven by inflammation, intestinal barrier protection, and microbiota-derived short-chain fatty acid signaling.

Furthermore, the combined use of butyric acid with other additives exerts a similar effect. Diether et al. [42] investigated the effect of adding MCOA at a dose of 2000 mg/kg on microbiota in the intestine of weaned piglets. The results showed that the abundance of *Lactobacillus* notably elevated. More interestingly, it was discovered that bile acid production increased [42]. These effects collectively improved the systemic metabolism of piglets and maintained the stability of the intestinal microbiota. Previously, Castillo et al. [75] found that adding 3000 mg/kg butyrate, avilamycin, and plant extracts to weaned piglet diets significantly enhanced ileal and cecal purine bases. It was also observed that the intestinal health of weaned pigs improved when 350, 700, 1050 mg/kg sodium butyrate was added to a diet containing benzoic acid [44]. These improvements were indicated by a reduced abundance of *Phascolarctobacterium*, *Campylobacter* and *Bacteroides*, as well as an increased abundance of *Prevotella*, *Megasphaera*, *Blautia*, *Streptococcus*, *Faecalibacterium* [44]. Furthermore, Zhong et al. [36] demonstrated that microbially derived butyrate regulated jejunal microbial homeostasis by increasing the abundance of *Erysipelotrichaceae*, *Lachnospiraceae*, *RF9_norank*, and reducing *Lactobacillaceae*, *Ruminococcaceae* during the first week after weaning. In summary, the above findings suggest that supplementing piglet diets with butyric acid and its derivatives’ can maintain intestinal health by increasing the abundance of beneficial bacteria, as well as reducing the abundance of harmful bacteria. A summary of butyrate as a functional feed additive for piglets is presented in Table 2 and Table 3. Also, a graphic abstract of butyric acid applications in piglet nutrition is shown in Figure 2. The detailed mechanism diagram of the effect of butyric acid on weaned piglets is shown in Figure 3.

## 4. Conclusions

In conclusion, numerous studies have explored the role of butyric acid and its derivatives as feed additives in weaned piglet nutrition, highlighting their multifaceted benefits. Butyric acid and its derivatives can be developed as promising functional feed additives for weaned piglets, offering a viable strategy to enhance growth performance, antioxidant activity, intestinal physiological functions, immune function. Based on current knowledge, butyric acid can be supplemented as a feed additive in pig diets at a dosage range of 350–3000 mg/kg, whereas higher supplementation dosages may exert adverse effects on animal productivity and health. However, in the healthy pig model, the dosage of the butyric acid additive depends on its different forms. The optimal dosage range for butyrate sodium is 1000–2100 mg/kg, while for butyrate glyceride, it is 1000–3000 mg/kg. In addition, in vitro experiments showed that butyric acid could have a positive effect on IPEC-J2 cells at a dose of 220–440 mM. This might be influenced by environmental challenges such as LPS, DON, ETEC, etc. Under the challenges of LPS, DON, ETEC, etc., the range of butyric acid additive dosage is 1000–3000 mg/kg. Future research efforts should further investigate the optimal dosage and application strategies of butyric acid for weaned piglets, as well as the specific mechanisms underlying the additive effects when combined with other feed additives. This is of great significance for moving toward a more sustainable and antibiotic-free pig production future.

## Figures and Tables

**Table 1:** A comparison of main characteristics of different butyric acid forms.

Forms	Stability	Odor	Site of Release	Bioavailability	Applications	References
Sodium butyrate	Safe, stable and easy to store	Unpleasant	Stomach, duodenum, jejunum	The highest. It completely dissolves in water and releases 100% butyric acid	It can be used as an additive for weaned piglets’ feed to improve growth performance	[9,10,11]
Coated sodium butyrate (Chemically protected sodium butyrate and Encapsulated protected sodium butyrate)	Stable, with a relatively high recovery rate, but lower than that of ordinary sodium butyrate	Reduce odor	Ileum, cecum, colon	Very high. The special coating increased the proportion of butyric acid reaching the distal intestine compared to the free butyric acid	It can be specially selected with coating technology according to the requirements	[12,13,14]
Butyrate glycerides	The most stable and resistant to high temperatures	Almost odorless	Targeted release in specific intestinal segments	It cannot be fully released and requires lipase	It can be achieved by adding it to the drinking water to improve the growth performance of weaned piglets	[15,16,17]

**Table 2:** The summary of butyric acid and its derivatives as functional feed additives for healthy weaned piglets without challenged or specific stress models during the past twenty years.

Animals	Supplemental Dosage (mg/kg)	Form	Duration (Days)	Main Findings					References
				Growth Performance	Antioxidant Capacity	Inflammatory Response	Intestinal Morphology	Microbiota	
Weaned piglet (5.17 kg BW)	1500 (21–32 d) 1000 (32–45 d)	sodium butyrate	24	↑ ADG, final BW	Jejunum: ↑ *GPX*, *SOD*	-	Ileum: ↑ VH, ↓ Peyer’s patches	-	[10]
Weaned piglet (7.93 kg BW)	2000	sodium butyrate	28	↑ ADFI	Plasma: ↑ GSH-Px	Plasma: ↓ IL-8 Colon: ↓ *TLR4*, *IKKα*	-	-	[37]
Weaned piglet (5.8 kg BW)	2000	sodium butyrate	35	no effects	-	Ileum: ↑ *CLDN1*, *MUC1*, *PKC*, *ITGB*	↑ duodenal and ileal VH, ↓ jejunal and colonic CD, ↑ jejunal and ileal VH/CD	Colon: ↑ *Lactobacillus*, *Blautia*, *Eubacterium_rectale_group*, *Subdoligranulum*, *Coprococcus_3*; ↓ *Rikenellaceae_RC9_gut_group*, *Streptococcus*, *Prevotellaceae_NK3B31_group*	[64]
Weaned piglet (7.81 kg BW)	3000	sodium butyrate	28	↓ F/G (15–28 d)	-	-	Ileum: ↑ VH, VH/CD; ↓ cleaved caspase 3, ↑ Ki67 Jejunum: ↑ villin, cleaved caspase 3	-	[60]
Weaned pig (6.89 kg BW, trial 1; 4.70 kg BW, trial 2)	350, 700, 1050 (trial 1 and 2)	sodium butyrate	35	quadratically ↑ ADG, final BW (trial 1); linearly ↑ ADFI (trial 2).	-	-	-	↑ *Prevotella*, *Megasphaera*, *Blautia*, *Streptococcus*, *Faecalibacterium*; ↓ *Phascolarctobacterium*, *Campylobacter* and *Bacteroides* (trial 2)	[44]
Weaned piglet (8.5 kg BW)	450	sodium butyrate	14	↑ ADG	-	Jejunum: ↓ histamine, tryptase, TNF-α and IL-6 levels, *MCT7*, *TNF-α*, *IL-6* mRNA levels	Jejunum: ↑ VH, VH/CD; ↑ TER, ↓ FD4	-	[65]
Piglet (7-day-old)	3000	sodium butyrate	60	no effects	-	-	no effects; Jejunum: ↑ mass; Ileum: ↑ length;	Caecum chyme: ↑ propionic	[41]
Weaned piglet (6.0 kg BW)	3000	sodium butyrate	21	↓ F/G	-	-	↑ goblet cells in the colon	-	[61]
Weaned piglet (6.0 kg BW)	3000	sodium butyrate	21	-	-	-	-	Ileal and cecal bacterial activity: ↑ purine bases	[75]
Weaned piglet (24-day-old)	2000	chemically protected sodium butyrate	28	↑ ADG, ↑ ADFI, ↓ F/G	Serum: ↑ T-AOC, SOD, GSH-Px, CAT, ↓ MDA Colon: ↑ *Keap1*, *Nrf-2*, *CAT*, *SOD1*;	Serum: ↓ TNF-α, ↑ IL-10 Colon: *IL-10*, ↓ *IL-1β*	Colon: *Claudin-1*	Regulated	[12]
Weaned pig (7.1 kg BW)	2500, 5000	fat-protected butyrate	21	↑ ADG, ADFI, ↓ Bone mineral content (5000)	-	-	-	-	[76]
Weaned piglet (4.69 kg BW)	2000 (20–34 d), 1500 (34–48 d), and 1000 (48–69 d)	encapsulated sodium butyrate	49	↓ F/G				Caecum: ↓ *Streptococcaceae*, *Streptococcus*	[14]
Weaned piglet (6.68 kg BW)	1000, 2000, 4000	compound sodium n-butyrate (85% sodium butyrate)	42	no effects	-	-	-	Cecal chyme: linearly ↑ isobutyric acid concentration, pH	[77]

Abbreviation: ↑, increase; ↓, decrease; ADG, average daily gain; ADFI, average daily feed intake; BW, body weight; CAT, catalase; CD, crypt depth; CLDN1, claudin-1; FD4, fluorescein isothiocyanate dextran 4 kDa; F/G, feed/gain ratio; GSH-Px/GPX, glutathione peroxidase; IL-6/1β/8/10, interleukin 6/1β/8/10; IKKα, inhibitor of kappa b kinase alpha; ITGB, integrin subunit beta; MCT7, mast cell-specific tryptase 7; MDA, malondialdehyde; MUC1, Mucin1; PKC, protein kinase c; SCFA, short chain fatty acid; SGLT1, sodium-glucose linked transporter 1; SOD, superoxide dismutase; T-AOC, total antioxidant capacity; TER, transepithelial electrical resistance; TLR4, toll-like receptor 4; TNF-α, tumor necrosis factor-α; T-SOD, total superoxide dismutase; VFA, volatile fatty acid; VH, villus height; VH/CD, villus height/crypt depth; ZO-1, zonula occludens-1—means that the parameter was not detected or no relevant results were obtained under the experimental conditions.

**Table 3:** The summary of butyric acid and its derivatives as functional feed additives for weaned piglets with challenged or specific stress models during the past twenty years.

Animals	Supplemental Dosage (mg/kg)	Form	Duration (Days)	Main Findings					References
				Growth Performance	Antioxidant Capacity	Inflammatory Response	Intestinal Morphology	Microbiota	
Weaned piglet with LPS challenge (6.3 kg BW)	2000	sodium butyrate	28	-	-	Longissimus mRNA level: ↓ *IL-6*; Serum: ↑ cortisol after LPS infection	-	-	[38]
Weaned piglet challenged with LPS (9.10 kg BW)	3000	coated butyrate	21	-	-	Jejunum: ↓ IL-1β, IL-6, NF-κB, NF-κB p65, HIF-1α, ↑ IL-10, IL-13, TGF-β	Jejunum and ileum: ↑ VH, VH/CD Jejunum: ↓ apoptosis index, Caspase 3 mRNA level	-	[56]
Weaned piglet infected with *Salmonella* Typhimurium (8.3 kg BW)	2100	protected sodium butyrate	16	Behavior: ↓ lying laterally without contact in the afternoon	-	-	-	*Salmonella* shedders: ↓ fecal and colonic digesta	[13]
Weaned piglet infected with ETEC F4 and F18 (21~24-day-old)	1000	butyrate glycerides	14	↓ diarrhea frequency	-	Serum: ↓ TNF-α	-	-	[15]

Abbreviation: ↑, increase; ↓, decrease; BW, body weight; CD, crypt depth; HIF-1α, hypoxia-inducible factor 1 alpha; IL-6/1β/10, interleukin 6/1β/10; LPS, lipopolysaccharide; NF-κB p65, nuclear factor-kappa b p65 subunit; TGF-β, transforming growth factor-beta; VH, villus height; VH/CD, villus height/crypt depth—means that the parameter was not detected or no relevant results were obtained under the experimental conditions.

## Data Availability

Not applicable.

## Abbreviations

The following abbreviations are used in this manuscript:

ADFI	Average daily feed intake
ADG	Average daily gain
BW	Body weight
CAT	Catalase
CD	Crypt depth
CLDN1	Claudin-1
FD4	Fluorescein isothiocyanate dextran 4 kDa
F/G	Feed/gain ratio
GSH-Px/GPX	Glutathione peroxidase
HIF-1α	Hypoxia-inducible factor 1 alpha
IKKα	Inhibitor of kappa b kinase alpha
IL-6/1β/8/10	Interleukin 6/1β/8/10
IPEC-J2	Intestinal porcine epithelial cell line
ITGB	Integrin subunit beta
LPS	Lipopolysaccharide
MCT7	Mast cell-specific tryptase 7
MCOA	A blend of medium-chain fatty acids, butyrate, organic acids, and a phenolic compound
MDA	Malondialdehyde
MUC1	Mucin1
MYD88	Myeloid differentiation primary response 88
P-NF-κB p65	Phosphorylated nuclear factor-kappa b p65 subunit
PKC	Protein kinase c
PPARα	Peroxisome proliferator activated receptor alpha
SCFA	Short chain fatty acid
SGLT1	Sodium-glucose linked transporter 1
SOD	Superoxide dismutase
T-AOC	Total antioxidant capacity
TER	Transepithelial electrical resistance
TGF-β	Transforming Growth Factor-β
TLR4	Toll-like receptor 4
TNF-α	Tumor necrosis factor-α
T-SOD	Total superoxide dismutase
VFA	Volatile fatty acid
VH	Villus height
VH/CD	villus height/crypt depth
ZO-1	Zonula occludens-1
